# Adapting the WHO hand hygiene ‘reminders in the workplace’ to improve acceptability for healthcare workers in maternity settings worldwide: a mixed methods study

**DOI:** 10.1136/bmjopen-2023-083132

**Published:** 2024-09-17

**Authors:** Catherine Louise Dunlop, Claire Kilpatrick, Laura Jones, Mercedes Bonet, Benedetta Allegranzi, Vanessa Brizuela, Wendy Graham, Amy Thompson, James Cheshire, David Lissauer

**Affiliations:** 1 Institute of Metabolism and Systems Research, University of Birmingham, Birmingham, UK; 2 Department of Integrated Health Services, World Health Organization, Geneva, Switzerland; 3 Institute of Applied Health Research, University of Birmingham, Birmingham, UK; 4 UNDP/UNFPA/UNICEF/WHO/World Bank Special Programme of Research, Development and Research Training in Human Reproduction (HRP), Department of Reproductive Health and Research, World Health Organization, Geneva, Switzerland; 5 Department of Infectious Disease Epidemiology and International Health, London School of Hygiene & Tropical Medicine, London, UK; 6 Institute of Education for Medical and Dental Sciences, University of Aberdeen, Aberdeen, UK; 7 Mersey and West Lancashire Teaching Hospitals NHS Trust, Prescot, Merseyside, UK; 8 Malawi-Liverpool-Wellcome Trust Clinical Research Programme, Blantyre, Malawi; 9 University of Liverpool, Liverpool, UK

**Keywords:** infection control, public health, obstetrics, neonatology, puerperal disorders, safety

## Abstract

**Abstract:**

**Introduction:**

Hand hygiene is key in preventing healthcare-associated infections, but it is challenging in maternity settings due to high patient turnover, frequent emergencies and volume of aseptic procedures. We sought to investigate if adaptions to the WHO hand hygiene reminders could improve their acceptability in maternity settings globally, and use these findings to develop new reminders specific to maternity settings.

**Methods:**

Informed by Sekhon *et al*’s acceptability framework, we conducted an online survey, semi-structured interviews and a focus group examining the three WHO central hand hygiene reminders (‘your five moments of hand hygiene’, ‘how to hand wash’ and ‘how to hand rub’) and their acceptability in maternity settings. A convergent mixed-methods study design was followed. Findings were examined overall and by country income status. A WHO expert working group tested the integrated findings, further refined results and developed recommendations to improve acceptability for use in the global maternity community. Findings were used to inform the development of two novel and acceptable hand hygiene reminders for use in high-income country (HIC) and low- and middle-income country (LMIC) maternity settings.

**Results:**

Participation in the survey (n=342), semi-structured interviews (n=12) and focus group (n=7) spanned 51 countries (14 HICs and 37 LMICs). The highest scoring acceptability constructs were clarity of the intervention (intervention coherence), confidence in performance (self-efficacy), and alignment with personal values (ethicality). The lowest performing were perceived difficulty (burden) and how the intervention made the participant feel (affective attitude). Overfamiliarity reduced acceptability in HICs (perceived effectiveness). In LMICs, resource availability was a barrier to implementation (opportunity cost). Two new reminders were developed based on the findings, using inclusive female images, and clinical examples from maternity settings.

**Conclusion:**

Following methodologically robust adaptation, two novel and inclusive maternity-specific hand hygiene reminders have been developed for use in both HIC and LMICs.

**Strengths and limitations of this study**

- The methodology and sampling used allowed for the participation of diverse groups, with a breadth of expertise and professions, from a variety of settings and reflected a wide range of resource availability in maternity settings.
- The survey translation into four languages improved accessibility of participation.
- The study was limited by conducting the qualitative interviews and focus group only in English, despite the survey being available in four languages. It is possible that some respondents could have had difficulty in the interpretation of questions and expression of responses if their language of choice was not an available option in the survey or qualitative interviews.
- In the survey, missing data were noted for later questions; up to 23% for the final demographic questions. The survey being conducted online may have limited participation or completion by those without a good internet connection. However, despite this, we did get a wide spread of participants from low- and middle-income countries.
- The impact of the two new WHO hand hygiene reminders on behaviour compliance in maternity settings was not part of this study methodology and remains to be evaluated.

## Introduction

 Maternity settings present unique challenges for infection prevention and control (IPC) due to the presence of a high volume of bodily fluids, frequent aseptic procedures, rapid turnover of patients^1^ and susceptibility of pregnant women and neonates to infections.^2^ There is potential for severe or fatal consequences to both a woman and infant if IPC is not achieved in maternity settings.^3^,^5^

Hand hygiene is key in preventing healthcare-associated infections (HCAI) globally,^6 7^ and helps prevent antimicrobial-resistant infections.^8^,^10^ Despite its importance, hand hygiene remains suboptimal globally and is a particular challenge in low- and middle-income countries (LMICs).^1 7 11^ The necessity of effective and reliable hand hygiene in healthcare settings, as one key IPC measure, has been further emphasised in the wake of the COVID-19 pandemic.^12 13^

Reminders in the workplace are part of the WHO multimodal improvement strategy (MMIS) strategy,^14^,^16^ an evidence-based approach, to support the improvement of hand hygiene in healthcare settings.^17 18^ Reminders are critical to influencing behaviours and are an established part of behaviour change approaches.^6 19 20^

There are three original and central WHO ‘reminders in the workplace’ (online supplemental materials 1).^21^,^23^ Two of the central reminders detail the steps in how and when to handwash^21^ or handrub.^22^ The third reminder entitled ‘Your 5 moments of Hand Hygiene’^23^ details five indications for healthcare workers to perform hand hygiene focused on the ‘patient zone’. These moments are ‘before touching a patient’, ‘before a clean or aseptic procedure’, ‘after body fluid exposure risk’, ‘after touching a patient’ and ‘after touching patient surroundings’.^6 23 24^ The patient zone is defined as ‘the patient and some surfaces/items in their surroundings that are temporarily and exclusively dedicated to them’.^25^ The patient zone is typically depicted with an adult patient in a bed illustrated inside it. Additionally, specific reminders demonstrate the times for hand hygiene in common or complex clinical situations, or those that may be particularly high risk to the patient or healthcare worker. Prior to our work, no reminders existed specific to maternity settings.

We sought to investigate if the original, central WHO hand hygiene reminders for the workplace were acceptable to healthcare workers in maternity settings, and if needed, to use these findings to develop new hand hygiene reminders specific to maternity settings.

## Methods

### Overall study design

We used a convergent, mixed methods study design^26^ to investigate the acceptability of the three original, central WHO hand hygiene reminders, for use in maternity settings globally. We characterised and explored acceptability using Sekhon *et al*’s theoretical framework, which defines acceptability as ‘a multi-faceted construct that reflects the extent to which people delivering or receiving a healthcare intervention consider it to be appropriate, based on anticipated or experienced cognitive and emotional responses to the intervention’.^27^ It consists of seven component constructs: affective attitude, burden, perceived effectiveness, ethicality, intervention coherence, opportunity costs and self-efficacy.^27^ Definitions of each construct in relation to this study context are included in online supplemental material 2.

Data collection included an online survey (online supplemental material 3), in-depth semi-structured interviews (online supplemental material 4A) and a focus group (online supplemental material 4B). Quantitative and qualitative data collection and analysis methods incorporated all seven constructs of acceptability to facilitate the mixed methods integration and interpretation of results.

### Quantitative survey

Expert sampling was used to recruit participants for the survey. It was sent via email to the GLOSS (Global Maternal Sepsis Study) network, which has a coverage of 54 countries and a range of healthcare professionals, researchers and data collectors.^28^ The snowballing method was used to promote recruitment.

The survey used Likert scales of 1–5 (1 indicating completely unacceptable and 5 indicating excellent acceptability for that construct). For some of the constructs, more than one question was included in the online survey to investigate this component of ‘acceptability’, in order to capture the full meaning of the construct in the responses. For some questions, free-text responses were also included for participants to add further detail. The survey was developed in English and translated into French, Spanish and Russian. The survey data collection was open for 6 weeks and 5 days, from the 26 April to 12 June 2018.

Quantitative data were analysed by acceptability construct and World Bank country income status,^29^ on Stata V.14. Where a construct of acceptability was represented by more than one question in the survey, the responses from these questions were analysed together. The median Likert for each acceptability construct was calculated for the overall survey population, and by country income status (high-income country (HIC) and LMIC). The t-test was used to assess for a difference between the proportions of good and excellent score in HIC and LMICs. This was performed to assess for differences in acceptability in different settings, where a ‘good’ score indicated a Likert of 4 and ‘excellent’ indicated a Likert of 5. HIC and LMIC settings were chosen for comparison because of a statistically significant difference in the odds of participants reporting reliable access to hand hygiene infrastructure from the survey responses. Proportions of good and excellent acceptability Likert scores were also calculated overall and by country income status.

### Qualitative interviews and focus group

The semi-structured interviews were conducted with practicing, qualified healthcare workers and public health practitioners with experience working in maternal health and purposively sampled to ensure a maximum variation sample by income setting and profession. Participants were approached through the team’s personal contacts at the University of Birmingham or were stakeholders in a maternal infection and sepsis study in Malawi.^30^ The focus group was conducted with a separate population of participants, to help further explore and develop the themes arising from the qualitative interviews, and how these could apply to newly developed hand hygiene reminders. This included GLOSS regional coordinators and key stakeholders in global maternal health representing a variety of maternity settings. All qualitative interviewees and focus group participants took part in English and were conducted face-to-face.

The interviews and focus group discussion were used to explore acceptability and to elaborate on complexities such as cultural dress requirements which were difficult to capture in the survey questions, and continued until data adequacy was reached. Interviews and the focus group were audio recorded and transcribed verbatim with anonymity maintained. The qualitative interviews and focus group took place between May and July 2018.

Qualitative data, including survey free-text answers, were analysed using the framework approach to thematic analysis,^31^ using a pragmatic paradigm^32^ supported by NVivo V.12. The seven acceptability constructs, plus an additional code of ‘other’ were initially deductively applied to the transcripts. ‘Other’ was used where findings did not obviously fit into any of the seven acceptability constructs. Following this, inductive themes were interpreted within each of the predefined constructs. The primary researcher was known to the interviewees. The researcher had clinical experience in obstetrics and gynaecology in the UK, and research experience in LMICs. Their intention was to understand the experience of working as a healthcare worker in the included settings, and how this experience impacted the practice of hand hygiene.

Using analyst triangulation, a second analyst independently reviewed and coded 10% of the transcribed data. The two analysts then met to confirm or refute the coding and themes developed, and agree on the final interpretative framework. The agreed approach was then taken for the remaining transcripts by the first analyst.

### Data integration

The quantitative and qualitative results were then convergently integrated and interpreted within each of the seven constructs to draw recommendations to improve the acceptability of the hand hygiene reminders for global maternity settings by the first analyst. These were presented in a joint display table.^33^

### WHO working group

These results were presented at a meeting at WHO headquarters to experts in IPC and maternity settings in May 2019 in a newly formed WHO working group comprising WHO and external academic staff. Based on the findings of the integrated mixed-methods results, a decision was made to adapt the existing reminders for maternity settings. The working group discussed the results of the mixed-methods study in several iterative meetings, refining the results and reaching core recommendations for change to improve the acceptability of the reminders for maternity settings. In summary, these core recommendations were developed from the study findings and refined by cross-checking with evidence from the literature, or using the expert working group opinion where evidence was not available.

### Development of new reminders

Finalised core recommendations were taken forward to poster level in several meetings with a WHO-contracted designer. Two new reminders were developed specific to maternity settings in a further iterative process with the working group and designer.

Participation in the online survey was voluntary and responses anonymous. A statement describing presumed consent was included on the first page of the survey if the participants completed the survey. Written, informed consent was obtained from all interviewees and focus group participants.

### Patient and public involvement

No patient involved: as this study focused on healthcare workers perception of the acceptability of the hand hygiene reminders, patients or the public were not included in the research methods.

## Results

### Participants demographics

In total, 342 professionals participated in the survey, 12 in the interviews and 7 in the focus group. Characteristics of participants are presented in table 1.

**Table 1:** Summary of all participants

		Survey*	Interviews and focus group
Job description	Public health practitioner or researcher in the field of maternal health	77	12
	Healthcare worker with experience working in maternity settings	166	18
	Infection prevention and control specialist	22	1
	Healthcare worker in GLOSS† facilities	100	7
	Other	7	1
Language	English	127	19
	Spanish	150	0
	Russian	41	0
	French	24	0

* Participants could select more than one job role.

† WHO Global Maternal Sepsis Study.

GLOSSGlobal Maternal Sepsis Study

Overall, participants represented 51 countries (14 HICs and 37 LMICs) (online supplemental material 5). The hand hygiene infrastructure available to participants is presented in online supplemental material 6. Participants from LMICs were statistically less likely than those in HICs to have access to reliable hand hygiene infrastructure, including running water, soap and alcohol-based handrub and these findings were statistically significant.

### Integrated findings of the original, central reminders’ acceptability for use in maternity settings

Of the survey participants, 91% stated they had seen the posters before and 78% stated that they were displayed in their workplace. All of those participating in the semi-structured interviews and focus groups stated they had seen the posters before.

A joint display summary of these integrated results by component construct is presented in table 2. The median Likert scores, and percentage of good (Likert score 4) and excellent (Likert score 5) scores, for each acceptability construct are presented. For each construct, these are presented for the survey population overall, by country income status and with an illustrative quotation. Findings for the three reminders were grouped together because of similarity in results and themes arising, to avoid duplication. However, where findings were specific to an individual reminder, this is specified.

**Table 2:** Joint display of mixed methods results for the three central hand hygiene reminders

Construct of acceptability^23^ (n)	Overall (n-342)		HIC* (n=76)†		LMIC‡ (n=187)†		Difference between proportions of good and excellent scores in HIC* and LMIC‡ (t-test p value)	Illustrative quotation from interviews/focus group
	Median Likert (IQR)¶	Good or Excellent scores§ n (%)	Median Likert (IQR)¶	Good or Excellent scores§ n (%)	Median Likert (IQR)¶	Good or excellent scores§ n (%)		
Intervention coherence** (684)	5 (4–5)	623 (91)	5(4–5)	133 (88)	5(4–5)	349 (93)	0.0290	‘I think that everyone would know what to do when they see the posters. Like I said the message is clear, so reading through that and with the pictures attached everyone would know when to wash hands and how to do it*.*’ P2 Interviewee (LMIC)
Perceived effectiveness†† (991)	4 (3–5)	714 (72)	4 (3–5)	137 (60)	4 (4–5)	430 (77)	<0.0001	‘The messaging here is very clear as to how to do it, not why you need to do it. But I think why you need to do it also needs to be communicated.’ P12 Interviewee (HIC)
Burden‡‡ (307)	4 (3–5)	182 (59)	4 (3–5)	46 (61)	4 (3–5)	115 (62)	0.8835	‘In an emergency setting or in a pressured environment where you have to attend multiple places, probably I will still end up washing my hands or doing a hand rub, but am I actually going to do all the steps on this? Unlikely.’ P10 Interviewee (HIC)
Self-efficacy‡‡ (307)	5 (4–5)	246 (80)	5 (4–5)	62 (82)	5 (4–5)	154 (82)	0.8819	‘Everybody has got a hand to do, a role to play in the preventing sepsis and infection, worldwide that is, everybody has got a role to play. If someone doesn’t do it, we have a problem there.’ P3 Interviewee (LMIC)
Opportunity cost‡‡ (307)	5 (3–5)	230 (75)	4 (3.5–5)	57 (75)	5 (4–5)	144 (77)	0.7284	‘By doing hand hygiene you tend to lower the cases of maternal infection and maternal sepsis thereby lessening your job.’ P3 Interviewee (LMIC)
Ethicality‡‡ (307)	5 (4–5)	258 (84)	5 (4–5)	62 (82)	5 (4–5)	160 (86)	0.4196	‘It’s already part of the work because we have to do the right things and the infection prevention is one of the right things which we are supposed to do.’ P7 Interviewee (LMIC)
Affective attitude (342)	4 (3–5)	214 (63)	4 (3–5)	50 (66)	4 (3–5)	111 (59)	0.3319	‘I am thinking that for the picture it would be good to have a pregnant woman, her picture, because that would relate directly to the department that you are referring to, and for our setting we really like to have pictures that have a meaning for what exactly we are trying to achieve.’ P2 Interviewee (LMIC)

* High- income countries.

† For analysis by country income status, data is excluded if country unknown.

‡ Low or middle- income countries.

§ Good or excellent scores represent a score of 4 (Ggood) or 5 (Eexcellent) on the lLikert scale.

¶ Interquartile rangeIQR.

** Two -component questions reflected to define this construct.

†† Three -component questions reflected to define this construct, 3% missing data.

‡‡ 10% missing data.

HIChigh-income countryLMIClow- and middle-income country

The constructs that scored highest for the acceptability of the hand hygiene reminders were clarity of the reminders (intervention coherence), participants’ confidence in their performance of hand hygiene based on the reminders (self-efficacy) and the alignment with their personal values (ethicality). The lowest performing acceptability constructs were the perceived difficulty of performing hand hygiene based on the reminders (burden) and how the reminders made the participant feel (affective attitude).

Additional inductive themes were interpreted within several of the constructs, which influenced perceived acceptability including: the desire for evidence; benefits of pictorial instructions; uncertainty about the intended audience, participant sense of duty and desire to protect patients; impact of team culture; the importance of being context (maternity and location) specific and visually appealing; and lastly challenges of workload, resource availability and time. Overfamiliarity of the reminders was an issue for participants from HICs. In LMICs resource availability and its impact on implementation was a concern as the reminders were not felt to be reflective of the realities of a resource-limited setting.

The integrated mixed-methods findings as presented in table 2, are reported below, set out against each acceptability construct.

#### Intervention coherence

There was a good understanding of the intervention in both quantitative and qualitative findings, with a median Likert score overall of 5 (IQR 4–5), as shown in table 2. Pictures demonstrating how to perform each step were reported as helpful, as in the ‘How to Handrub’ and ‘How to Handwash’ reminders, particularly where English was not the first language, or literacy not universal.

I think the fact that it’s so clearly laid out, the pictures are very straightforward, there’s no noise to the poster, it’s very logical, the arrows, the colours, the diagrams, it’s all straightforward. It’s all very logical, very easy to follow. P11 Interviewee (LMIC)

In the qualitative interviews and focus group, it was not well understood what would constitute each hand hygiene opportunity in a maternity setting. Clarity was specifically requested for what would constitute a clean or aseptic procedure in a maternity setting.

But I also think that having some examples, for example before clean and aseptic procedure you could just have some pictures that show what a clean and aseptic procedure is in a maternity ward. P2 Interviewee (LMIC)

#### Perceived effectiveness

The reminders were thought to be successful at promoting hand hygiene in the survey with a median Likert score of 4 (IQR 3–5). In LMICs, 77% of participants scored good or excellent but only 60% from HIC which was statistically significant (p<0.0001).

The qualitative responses did not confirm these quantitative results. It was felt that the reasons to practice good hand hygiene were missing from the reminders.

But the biggest thing for me is the lack of any motivation which comes from just some way of presenting why it is actually important to do this. P1 Interviewee (HIC)

Overfamiliarity was an issue in HICs.

These posters are so ubiquitous I barely notice them anymore. Survey respondent (HIC)

#### Burden

The burden of engaging with the intervention was a lower performing construct with 59% good or excellent scores on the Likert scale. The median Likert was 4 (IQR 3–5). This was concordant in the qualitative findings. The steps required for effective hand hygiene, as well as the 5 moments themselves, were perceived as too time consuming to complete in full, due to the frequencies of emergencies in maternity settings.

It’s essentially making a judgement call between two harmful situations, so the harm that I may introduce by not washing my hands properly vs the harm of not attending a pathological trace [fetal heart monitor reading]. P10 Interviewee (HIC)

Additionally, in LMICs for some participants the availability of hand hygiene resources affected the burden of engaging in the intervention. However, other respondents reported a low burden in engaging in effective hand hygiene, perceiving it as easy and quick.

It’s very easy…we do it, it’s our habit. P4 Interviewee (LMIC)

#### Self-efficacy

Survey participants had confidence they could perform the behaviours required to achieve hand hygiene. The median Likert response was 5 (IQR 4–5). This was mostly confirmed in the qualitative findings. The pictorial representation of steps and opportunities for hand hygiene had the biggest impact on self-efficacy. Self-efficacy reportedly could be improved further through pictorial representation of examples specifically relevant to maternity settings.

However, the time taken to hand wash (linked to burden construct) also impacted on self-efficacy. This included competing with the number of patients and volume of emergencies.

But when things get too much, I don’t think it would be 100% following the actual way of washing hands. P2 Interviewee (LMIC)

#### Opportunity costs

Survey participants did not anticipate a high cost to other aspects of their role because of engaging with the intervention. The median Likert scale was 5 (IQR 3–5). This was mainly confirmed in the qualitative findings. Frequently respondents reported positive impacts from engaging in the intervention.

We are protected, the patients are protected, we as a health worker as well are protected, so it’s the goodness for both of us, yeah. P6 Interviewee (LMIC)

However, there was a concern about not being able to manage other duties if hand hygiene was adhered to.

I mean, as a junior doctor, if you are washing your hands every single time you come into contact with a patient environment you will be spending most of your day washing your hands and not doing what you need to do. P1 Interviewee (HIC)

#### Ethicality

Survey responses indicated that hand hygiene was in keeping with participant’s value system, with a median Likert of 5 (IQR 4–5). This was mainly confirmed in the qualitative findings. There was a commonly felt duty of care as healthcare workers to protect patients.

These posters help me to do the hand washing or hand rub properly with the intention that bearing in mind that I am protecting somebody nearby, a mother and a baby, that is it, the future world. P3 Interviewee (LMIC)

The importance of the reminders being representative and reflecting the specific maternity environment was emphasised. Including a woman as the patient in the ‘Your 5 moments of hand hygiene’ reminder was commonly mentioned in both the survey and interviews.

It could be maybe a pregnant lady [in the reminder] pleading saying if you don’t wash hands, I am five times more likely to die of sepsis. P10 Interviewee (HIC)

Cultural dress requirements were explored, and most of the survey and qualitative participants did not request specific clothing if a female patient was depicted. Participants could indicate multiple options in this part of the survey, but 217 responded that no specific dress requirements were needed for their setting. 73 requested for female legs to be covered, 24 for hair to be covered, 19 for arms to be covered and 9 for her face to be covered.

#### Affective attitude

How the reminders made participants feel was one of the lowest-scoring constructs in the survey with median Likert score of 4 (IQR 3–5). This was concordant with the qualitative interviews and focus group. Several participants reported feeling guilty when they failed to comply with hand hygiene. A sense of apathy regarding the reminders and hand hygiene was also present. The reminders were reported as overfamiliar and ‘grey’ (P13 (HIC)). Some were actively disengaged from the intervention because the images did not appear to apply to them.

It looks like an ITU [Intensive Therapy Unit, also known as Intensive Care Unit], and I don’t work in an ITU so it’s not my business. P14 Focus group participant (LMIC)

The importance of the reminders being context-specific to maternity and cultural settings, with local role models was reported to improve compliance.

We see a smiling woman or a smiling mother or anything like that, a lot of people get encouraged and they really want to be part of it. P2 Interviewee (LMIC)

#### Other

There were two inductively interpreted themes from the qualitative data, patient involvement in the hand hygiene and glove usage. Several participants requested that the reminders and hand hygiene intervention should address patients and visitors, as well as healthcare workers.

I asked the question whether this poster is for healthcare providers only or for healthcare providers and relatives. I think that they are required for both. P18 Focus group participant (LMIC)

Additionally, many participants requested additional guidance in the reminders on glove usage. Appropriate indications for glove use were considered an important omission in the current reminders, as gloves are used regularly in maternity settings and in low-income settings availability may be scarce.

I think most of the people in our country have used gloves as a backup method whereby with the water issues that are there most people just prefer to have gloves on. P2 interviewee (LMIC)

### Recommended adaptions for maternity settings

The integrated findings suggested changes to the hand hygiene reminders, specifically the ‘Your 5 moments’ reminder, which would improve acceptability for maternity settings. Quantitatively, 33% of participants were unsure or thought that changes could be made to the ‘Your 5 moments’ reminder to improve acceptability in maternity settings, which decreased to 28% for the ‘How to handwash’ reminder and 25% for the ‘How to handrub’ reminder. This was discordant with the qualitative results. The majority felt strongly that changes to the reminders would improve their acceptability in maternity settings. As with the survey results, more changes were felt to be helpful in the ‘Your 5 moments’ reminder than in the ‘How to’ reminders.

The recommendations for adaptions relevant to maternity settings from the integrated findings are presented in table 3, separated into acceptability constructs.

**Table 3:** Summary of recommended adaptions to the reminders to increase acceptability for use in maternity settings

Acceptability construct	Quantitative recommendations (number of participants making this suggestion)	Qualitative recommendations to increase acceptability in maternity settings
Intervention coherence	Steps shown to perform good hand hygiene (10)	Include an example of a clean or aseptic procedure specific to maternity settings.Explain the hand hygiene action should occur ‘just before’ the opportunity.
Perceived effectiveness	Improve text content (26)Colour scheme (43)	Explanation of benefits and evidence for hand hygiene.Eye catching colour scheme with bolder, bigger text.Clarify intended audience.Focus on high-impact opportunities for the prevention of HCAI specific to maternity settings.
Burden	Explanation of the benefits of good hand hygiene (29)	Evidence and reasoning for composite intervention—5 moments and 12 steps.Address barrier of ‘time’.
Self-efficacy	Healthcare workers shown (22)	Using pictures to show opportunities specific to maternity settings, rather than text.Promoting teamwork.
Opportunity costs	Reduce volume of text (33)	Reduce volume of text.Emphasise speed of hand washing action.
Ethicality	Patient shown (39)Equipment shown (21)	Representing local environment through local language use and ethnicity of patient.Female patient in the central image.Reference healthcare worker desire and sense of duty to protect patients.
Affective attitude	Style of drawings (44)	Modernise language and style of illustrations.Images representative of maternity environments.Local people demonstrating good practice, and acting as role models in the reminders.
Other	Other (21)No changes (2)	Include patients and relatives in intended audience.Additional information about glove use.

HCAIhealthcare-associated infections

### Development of the new hand hygiene reminders for maternity settings

Based on these integrated findings, a working group including the WHO, IPC and maternal health experts and academic partners developed core recommendations for change to improve the acceptability of the hand hygiene reminders for maternity settings. These were; relevant images, infographics instead of text, emphasis on hand hygiene around aseptic procedures, reducing the hand number of moments, reducing text volume and highlighting the importance of hand hygiene. Other specific recommendations were also incorporated in the reminder design such as updating the colour scheme to make the reminders more eye catching, modernising the illustrations and including information about glove use. As six of the seven acceptability constructs showed no statistically significant difference between HIC and LMICs, the reminders were developed for use in both settings.

The images needed to be relevant to maternity settings and inclusive of maternity settings with a variety of resource availability. Therefore, a collective decision was made to include the woman and her neonate in the picture, with no clinical equipment. An updated colour scheme was used with modernised images. The woman depicted was designed to look relevant for multiple country settings with a non-specific ethnicity and dressed conservatively. She was also depicted to be smiling and well, rather than in the sick role. For microbiological purposes, the mother and her newborn baby remain a part of the same patient zone^34^ and has been studied to address the application of the 5 moments in these circumstances.^35^ It is therefore acceptable for these patients to share the same bed and the new hand hygiene reminders were drawn with the mother and newborn in the same patient zone to reflect this. Hand hygiene actions as per the 5 moments can be applied in such circumstances.

A table of hand hygiene moments and rationale specific to maternity settings was iteratively developed using the working group to choose the examples most relevant to maternity settings for the new reminders. This table is included in online supplemental material 7. The working group selected examples from this table with the most evidence in the literature for reducing infection in maternity settings to include in the new reminder. Consensus was used where evidence was not available. In this way, examples of aseptic procedures in maternity settings, as recommended, were highlighted on the reminders.

One new reminder was developed with the table of explanation from the original, central reminders explaining the moments of hand hygiene. This table had a row for each moment to explain why each moment was needed, to address the decision to emphasise the importance of hand hygiene. To simultaneously address the decision to reduce text volume, an additional reminder was developed with just the central image and bullet point examples of maternity scenarios related to each hand hygiene moment. A novel statement on hand hygiene around glove use was included on both reminders.

Due to WHO requirements for the new reminders and the complexity of including multiple aspects on one reminder, it was decided by consensus of the working group that it was not possible to include infographics to depict hand hygiene moments in this iteration, although it was agreed this will be considered in future reminders. Due to evidence for the 5 moments of hand hygiene approach, it was also decided not to reduce the number of moments included.

**Figure 1:**
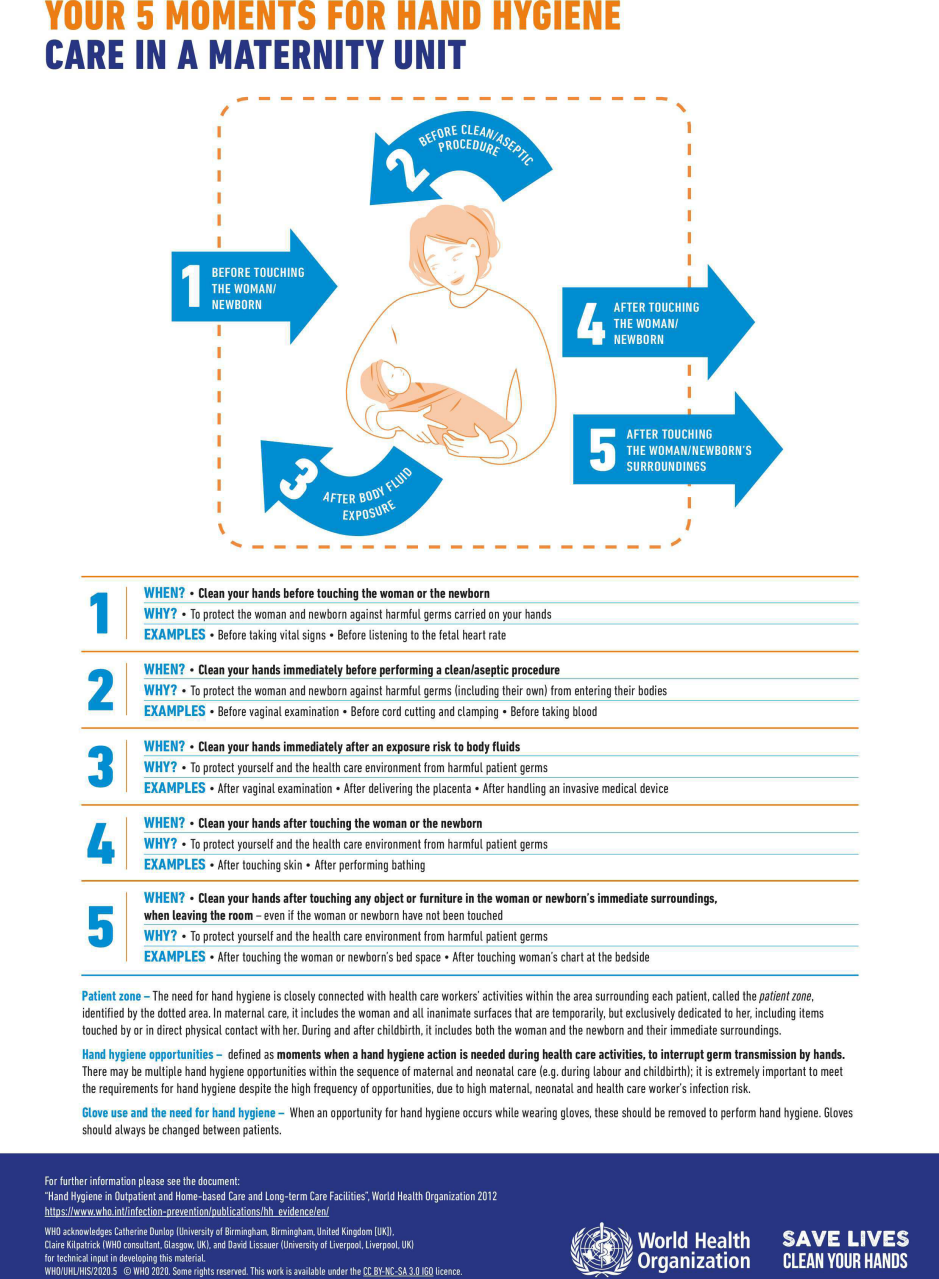
Your 5 moments for hand hygiene. Care in a maternity unit A.^57^

The two new reminders were launched on World Hand Hygiene Day in 2020, for the WHO year of the Nurse and Midwife. These are included in figures1  2: Your 5 moments for hand hygiene. Care in a maternity unit A and B.

**Figure 2:**
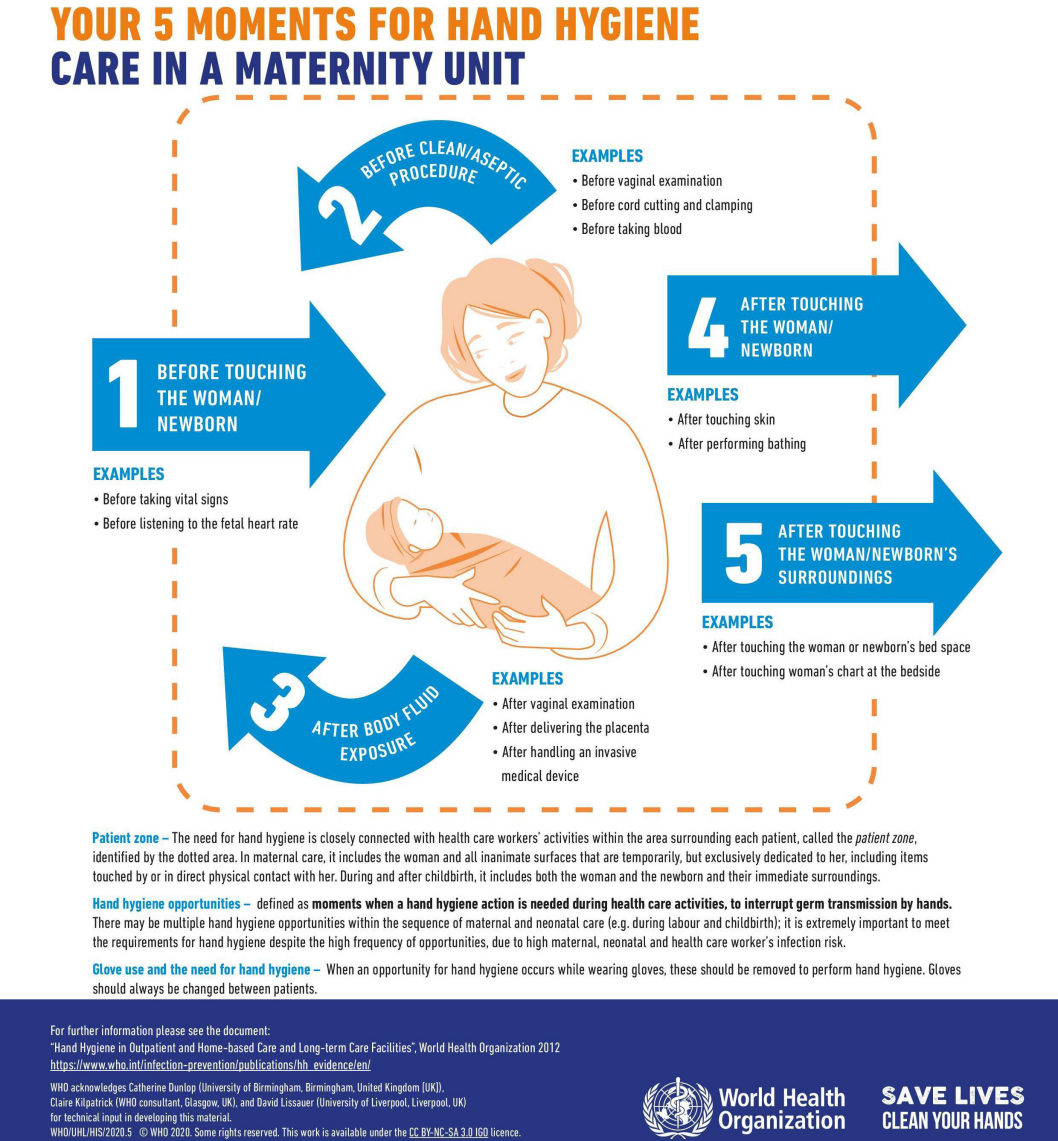
Your 5 moments for hand hygiene. Care in a maternity unit B.^58^

## Discussion

### Main findings

In this convergent, mixed-methods study we investigated the acceptability of the three original, central WHO hand hygiene reminders for use in global maternity settings. Participants found the reminders to be clear (intervention coherence), felt confident they could perform hand hygiene using the reminders (self-efficacy) and found them to be in line with their personal values (ethicality). However, the work (burden) of practicing hand hygiene was reported as high and participants had some negative perceptions about the reminders (affective attitude). Additionally, participants reported a: desire for evidence; the benefits of pictorial instructions; and the importance of being context-specific. From these findings, recommendations were drawn to improve the acceptability of the reminders for maternity settings.

An expert working group of WHO and academic IPC and maternal health professionals used these integrated findings to develop two new hand hygiene reminders specific to maternity settings. The new reminders were designed to improve acceptability for maternity settings based on the study findings. These included an updated colour scheme, modernised illustrations, depicting a woman with her baby, highlighting the importance of hand hygiene and clarification around glove use. These new reminders have now been released by WHO and are available freely for public use.

### Strengths and limitations

The survey translation into four languages improved accessibility of participation. The participants crossed multiple professions, from variety of settings and reflecting a breadth of resource availability.

The study was limited by conducting the qualitative interviews and focus group only in English. The survey was more accessible, as it was available in four languages. However, it is possible that some respondents could have had difficulty in interpretation of questions and expression of responses if their language of choice was not an available option. In the survey, missing data were noted for later questions; up to 23% for the final demographic questions. The survey being conducted online may have limited participation or completion by those without good internet connection. However, despite this, we did get a wide spread of participants from LMICs. The study could have been limited by not including the perspective of patients or visitors, who also play an important role in effective IPC in healthcare settings. However, the reminders under study are primarily aimed at healthcare workers.

### Interpretation

These findings are in keeping with previous research on hand hygiene reminders.^19 36 37^ In our results, we found the clarity of the hand hygiene intervention as depicted in the reminders (the intervention coherence) to be good or excellent (91% overall). This was in part, demonstrated because participants found use of pictures helpful in understanding the intervention and preferred this to reading text. A study by Harrison *et al*, exploring acceptability of hand hygiene reminders for neonatal infection prevention in community settings in Uganda, also found that understanding of the intervention was good.^37^ They similarly reported participant preference for images over text explanations.^37^

However, the perceived effectiveness of the reminders in promoting the importance of hand hygiene was one of the lowest-scoring constructs (72% overall), together with affective attitude (63% overall) and burden (59% overall). This is in keeping with prior commentary on this topic, which suggests that the reminders focus on ‘telling’ viewers about, rather than ‘selling’ the importance of hand hygiene.^36^ Previous research has commented that messaging in the reminders could be framed in terms of gains of the procedure, and that more than ‘training messaging’ is required to change behaviours.^19^

We found that a sense of duty to protect oneself and patients were positive enforcements of hand hygiene (ethicality). Appealing to a sense of duty in the healthcare worker and individual value systems within the reminders has been previously recommended.^19^ Our study sample was additionally keen to clarify the role of patients and visitors in hand hygiene, and whether the reminders could be directed towards them. The role of patients has previously been explored to improve hand hygiene compliance^38^,^40^ and WHO does recommend patient involvement in hand hygiene promotion.^6^

Outside of the reminders, infrastructure was a key issue impacting on acceptability for many of the participants, which included access to water and other hand hygiene resources. This affected the self-efficacy and burden of engagement in hand hygiene practices. This issue has been found in other studies in maternity settings,^41^ in both a lack of running water^42^ and the absence of alcohol-based handrub.^43^ Access to alcohol-based handrub has been particularly associated with improvement in hand hygiene compliance,^43 44^ perhaps by reducing the burden and opportunity costs of hand hygiene.

In the newly developed reminders, the placement of a woman smiling in the central image was to reinforce the feeling of childbirth as a positive experience.^45^ This was done based on the findings in the ethicality and affective attitude constructs, to help clarify the intended audience of the reminders and to make them more acceptable for use by both healthcare workers and those receiving care. Additionally, aseptic procedures were particularly noted in the qualitative interviews as areas that should be addressed in maternity-specific reminders, because of the benefit in reducing HCAI. Aseptic procedures in global maternity settings are a particular risk for HCAI to the woman and neonate, for example, during vaginal examinations.^11 41 46^ Therefore, specific written examples of aseptic procedures were included in the newly developed posters based on these findings. Lastly, our findings confirmed that glove use alongside hand hygiene remains an area of uncertainty in clinical practice,^6 47^ especially in settings where resources may be scarce. Globally, this issue was further exacerbated during COVID-19.^48^ Therefore information on glove use, drawing on the WHO glove pyramid,^49^ was included in the newly developed reminders.

Based on our findings, future iterations of the reminders could make use of the reported benefit of infographics to improve the accessibility of the reminders. A reminder focusing solely on the role of hand hygiene around a key aseptic procedure in maternity settings could improve education on this point. Potential examples of aseptic techniques which could be addressed are included in online supplemental material 7. Making reminders explicitly relevant for patients and visitors in addition to healthcare workers could improve awareness and the perceived effectiveness of IPC in healthcare settings overall. Considering this construct (perceived effectiveness) was one of the lowest scoring at 72% overall, there is further room for addressing this area of acceptability. Lastly, emphasising the gains of hand hygiene in maternity settings could further improve the acceptability, through lessening the perceived burden and opportunity cost to healthcare workers.

However, we acknowledge the reminders are only one part of the wider WHO MMIS,^15^ which has demonstrated a positive impact on hand hygiene compliance.^18^ The three central reminders were originally intended by WHO to be instructional and educational,^50^ designed to have long-lasting relevance to clinical care. Motivational messaging, as shown to be important in our findings and in the wider literature,^19 36^ is currently used in the WHO SAVE LIVES: Clean Your Hands World Hand Hygiene Day campaign,^51^ which each year aims to maintain the profile of hand hygiene. Motivational messaging in a hand hygiene reminder may be more suitable for an annual campaign poster, as key issues or eye-catching statistics may change based on time or setting. This may improve acceptability in HICs where posters were reported as ubiquitous and so became ‘unseen’. Future evaluation and innovations in promoting hand hygiene as part of an MMIS may also improve compliance, including automation of monitoring processes and feedback, or forcing functions.^52^ As a positive team culture was reported to improve self-efficacy, this could be a focus for future interventions in this field.

The issue of how reminders, as one part a wider strategy to motivate behaviour change,^20^ have an ongoing impact on hand hygiene behaviours has been outlined by WHO.^6^ Work conducted in both Switzerland and England^53^ specifically informed guidance for the frequency of changing promotional messaging as well as replacing longer-term reminders; all of which can have an impact on overall acceptability. The resulting WHO hand hygiene self-assessment framework indicators^54^ state that auditing reminders for damage and replacement should ideally take place every 2–3 months.^40^ Although, the exact frequency of change is not known, monthly reminders were noted to be more effective than quarterly reminders to sustain practice change among direct care providers in residential care facilities.^55^ Hand hygiene promotion should additionally be undertaken with reminders other than the three, central, original ones presented in this study. Further work to understand the ongoing impact of all prevailing reminders on behaviour is worthy of further inquiry.

Hand hygiene continues to be key in preventing HCAI^6 7^ and HCAIs still lead to maternal and newborn sepsis, which are top causes of maternal and neonatal death globally.^2 56^ Future research should evaluate the impact of the two new WHO reminders on hand hygiene behaviour compliance in maternity settings. The perspective of patients and visitors could also be considered, and the context of varying resources that ultimately on impact on practice.

### Conclusion

The study has shown that the WHO MMIS ‘reminders in the workplace’ could be adapted to improve acceptability for use in global maternity settings. Two novel maternity-specific hand hygiene reminders have been developed, relevant to HIC and LMICs. We recommend for these to be introduced into maternity care settings worldwide. Further research will be required to understand if these developments are effective in leading to increasing hand hygiene compliance in maternity settings.

## supplementary material

10.1136/bmjopen-2023-083132 online supplemental file 1

## Data Availability

Data are available upon reasonable request.
