# Supplementary material for: Adapting the WHO hand hygiene ‘reminders in the workplace’ to improve acceptability for healthcare workers in maternity settings worldwide: a mixed methods study
Source: BMJ Open. 2024 Sep 17;14(9):e083132. doi: 10.1136/bmjopen-2023-083132 (PMC11409321; doi:10.1136/bmjopen-2023-083132)
Supplement: online supplemental file 1 [file bmjopen-14-9-s001.pdf]

Supplementary Material 1: Three original, central Reminders in the Workplace(21–23)

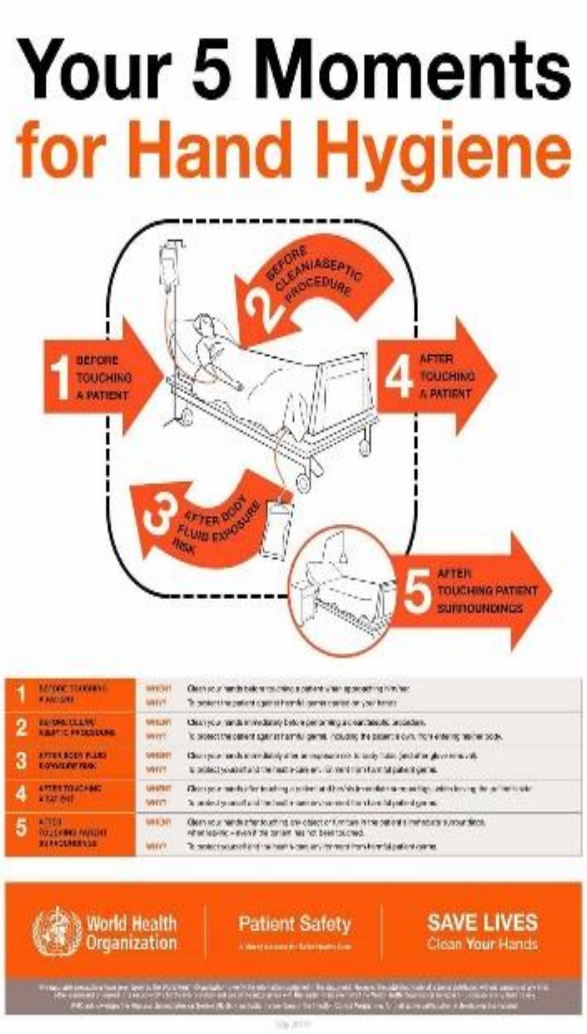

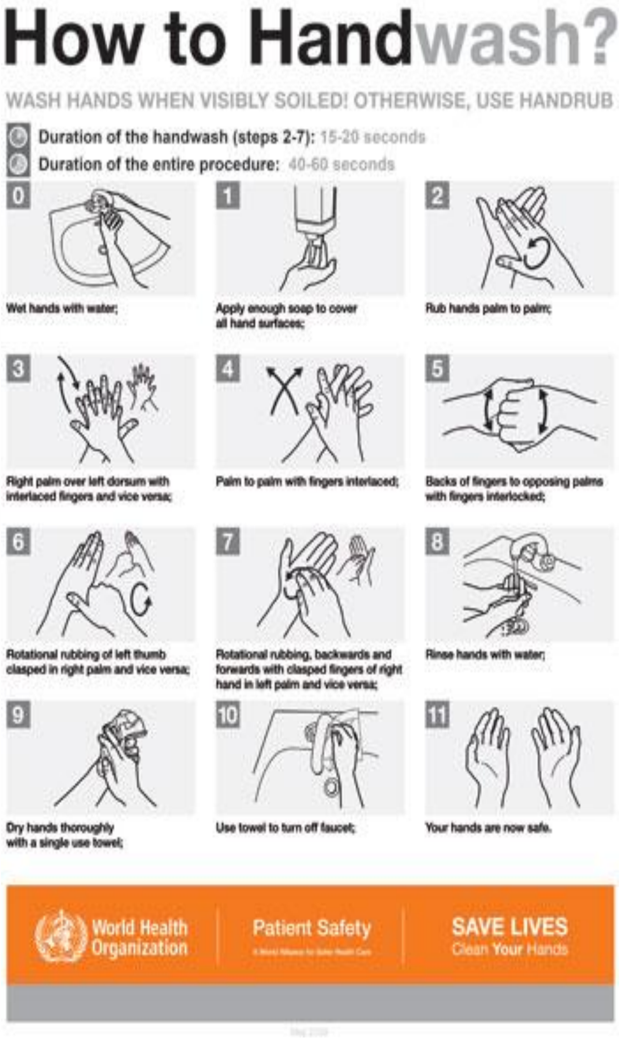

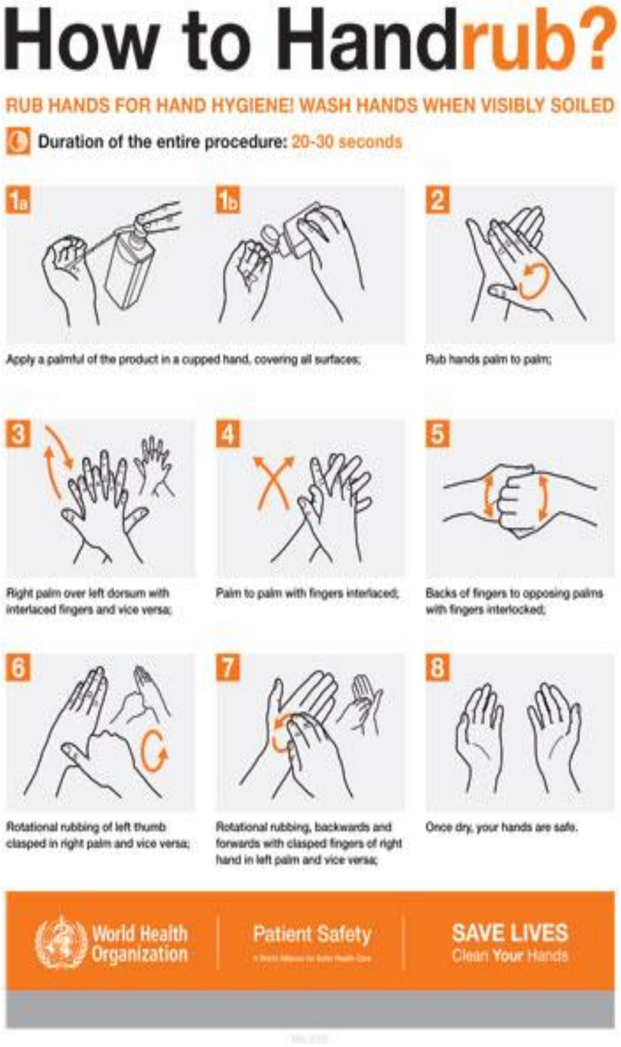

Supplementary Material 2: Seven constructs of acceptability by Sekhon et al(27)*, with definitions relevant to this study question*

Construct of Acceptability	Summary of definition
**Affective Attitude**	How the hand hygiene reminders made the person feel
**Burden**	The anticipated difficulty and effort of engaging with hand hygiene based on what was depicted in the reminders
**Ethicality**	The alignment of hand hygiene, as shown in the reminders, with the person's personal values
**Intervention coherence**	The clarity of the hand hygiene intervention as depicted in the reminders
**Opportunity costs**	Any additional consequences that occur for the person as a result of practicing hand hygiene, as it is depicted in the reminders
**Perceived effectiveness**	The person's perceived idea of how likely the reminders are is to achieve their purpose of increasing practice of hand hygiene
**Self-Efficacy**	The person's confidence in performing hand hygiene, as depicted in the reminders

Supplementary Material 3: Survey investigating the acceptability of the "WHO hand hygiene reminders in the workplace" for use in maternity settings

Introduction Emails:

Dear health care providers and maternal health experts,

A few months ago you responded to a similar survey for the GLOSS campaign. At the time you agreed to being contacted by our team in the future. We are now asking for your help with a further important issue.

As you know, hand hygiene is a critical step in preventing maternal infections and sepsis. We would like to get your feedback on the current WHO hand hygiene posters.

We are seeking opinions from those with experience working in a wide range of maternity settings to understand if the current general materials can be adapted to increase their acceptability and relevance to maternity settings around the world.

We would be grateful for your time in answering the following brief questions. We estimate this will take less than 15 minutes of your time. Your responses will be anonymous.

We encourage you to forward the following link to your colleagues at the healthcare facility where you work.

Link to the survey in English: **https://www.surveymonkey.com/r/hand_hygiene_materials**

If you have any questions or concerns about this survey, please feel free to contact: Dr. Catherine Dunlop, Research Fellow at University of Birmingham. Email: catherinedunlop@nhs.net

Thank you for your time completing this survey.

Survey on the WHO hand hygiene tools

The completion of this survey implies your consent to participate in our research on the WHO hand hygiene materials and how these can be adapted for maternity settings. All responses will be anonymous and used to inform future developments to these materials.

If you have any questions or concerns about this survey, please feel free to contact:

Dr. Catherine Dunlop, Research Fellow at University of Birmingham

Email: catherinedunlop@nhs.net

Thank you for your participation!

Please review the following posters, and answer the questions below.

"How to Handwash" poster [Image included]

"How to Handrub" poster [Image included]

"Your 5 moments of Hand Hygiene" poster [Image included]

Here are weblinks to the three posters. You may find it helpful to open them in another tab in your browser. We will be referring back to these posters throughout the survey.

How to handwash poster; http://www.who.int/gpsc/5may/How_To_HandWash_Poster.pdf?ua=1

How to handrub poster; http://www.who.int/gpsc/5may/How_To_HandRub_Poster.pdf?ua=1

Your 5 moments of hand hygiene poster;
http://www.who.int/gpsc/5may/Your_5_Moments_For_Hand_Hygiene_Poster.pdf?ua=1

1. Have you seen any of these posters before?

Yes, they are displayed at my place of work

Yes, I have seen them before but they are NOT displayed in my workplace

No, I have not seen these posters before

2. Overall, how clearly do the posters show what ACTIONS are required for good hand hygiene? [Intervention Coherence]

Not at all clearly				Extremely clearly
1	2	3	4	5

3. Overall, how clearly do the posters show WHEN it is necessary to wash or rub your hands? [Intervention Coherence]

Not at all clearly				Extremely clearly
1	2	3	4	5

4. Overall, how eye catching and noticeable are these posters? [Perceived effectiveness]

Not at all				Extremely
1	2	3	4	5

5. Overall, how clearly do these posters show the importance of good hand hygiene? [Perceived effectiveness]

Not at all clearly				Extremely clearly

1	2	3	4	5

6. Overall, how well do these posters represent maternity wards in facilities where you have worked? [Affective attitude]

Not at all representative				Completely representative
1	2	3	4	5

7. To what extent would these posters motivate you to perform good hand hygiene in maternity wards, in facilities where you have worked? [Perceived effectiveness]

Not at all				Strongly
1	2	3	4	5

8. How manageable do you think good hygiene is (as explained in the posters) in maternity wards, in facilities where you have worked? [Self-efficacy]

Not at all manageable				Completely manageable
1	2	3	4	5

9. How culturally appropriate are these posters to display in maternity wards, in facilities where you have worked? [Ethicality]

Not at all appropriate				Completely appropriate
1	2	3	4	5

10. If a pregnant woman was shown in a poster, what dress requirements are needed for cultural sensitivities in facilities where you have worked? Please tick all that apply [Ethicality]

Legs covered

Arms covered

Hair covered

Face covered

No specific dress requirements

Other (please specify):

11. How much effort would be needed to complete the tasks in the posters, in maternity wards in facilities where you have worked? [Burden]

Very high effort				Minimal effort
1	2	3	4	5

12. How difficult would it be for staff to complete other parts of their job if following all the instructions in these posters, in maternity wards in facilities where you have worked?

[Opportunity cost]

Very difficult				Not at all difficult
1	2	3	4	5

13. In regards to the "How to handwash" poster; do you think that this needs to be changed so that it is suitable for maternity wards in facilities where you have worked?

Yes

No

Unsure

14. What changes would you make to the "How to handwash" poster to encourage improvements in hand hygiene in maternity wards, in facilities where you have worked? Please tick all that apply:

Text content

Volume of text

Colour scheme

Style of drawings

Equipment shown

Steps shown to perform good hand hygiene

Patient shown

Healthcare worker shown

Explanation of the benefits of good hand hygiene

No changes

Other (please specify):

15. In regards to the "How to handrub" poster; do you think that this needs to be changed so that it is suitable for maternity wards in facilities where you have worked?

Yes

No

Unsure

16. What changes would you make to the "How to handrub" poster to encourage improvements in hand hygiene in maternity wards, in facilities where you have worked? Please tick all that apply

Text content

Volume of text

Colour scheme

Style of drawings

Equipment shown

Steps shown to perform good hand hygiene

Patient shown

Healthcare worker shown

Explanation of the benefits of good hand hygiene

No changes

Other (please specify):

17. In regards to the "Your 5 moments of hand hygiene" poster; do you think that this needs to be changed so that it is suitable for maternity wards in facilities where you have worked?

Yes

No

Unsure

18. What changes would you make to the "Your 5 moments of hand hygiene" poster to encourage improvements in hand hygiene in maternity wards, in facilities where you have worked? Please tick all that apply

Text content

Volume of text

Colour scheme

Style of drawings

Equipment shown

Steps shown to perform good hand hygiene

Patient shown

Healthcare worker shown

Explanation of the benefits of good hand hygiene

No changes

Other (please specify):

Please review the following posters, and answer the questions below.

"It's in your hands– prevent sepsis in health care" Main advocacy poster [Image]

"It's in your hands– prevent sepsis in health care" Healthcare worker poster [Image]

These appear as a part of a new WHO campaign on hand hygiene to prevent sepsis in healthcare facilities.

Here are the weblinks to these posters. You may find it helpful to open them in another tab in your browser. We will be referring back to these posters during the rest of the survey.

Advocacy poster; http://www.who.int/infection-prevention/campaigns/clean-hands/Advocacy2018.pdf?ua=1

Healthcare worker poster; http://www.who.int/infection-prevention/campaigns/clean-hands/HealthWorkers.pdf?ua=1

19. Overall, how eye catching and noticeable are the new campaign posters compared to the previous three posters? [Perceived effectiveness]

More eye-catching and noticeable

Less eye-catching and noticeable

About the same

20. If you would like to, please provide an explanation for your answer:

21. Overall, how clearly do the new campaign posters show the importance of good hand hygiene compared to the previous three posters? [Perceived effectiveness]

Better at conveying the importance

Worse at conveying the importance

About the same

22. If you would like to, please provide an explanation for your answer:

23. How well do the new campaign posters represent maternity wards in facilities where you have worked, compared to the previous three posters? [Affective attitude]

More representative

Less representative

About the same

24. If you would like to, please provide an explanation for your answer:

25. How culturally appropriate are the new campaign posters for maternity wards in facilities where you have worked, compared to the previous three posters? [Ethicality]

More culturally appropriate

Less culturally appropriate

About the same

26. If you would like to, please provide an explanation for your answer:

27. Overall, how do the new campaign posters compare to the previous three posters in making good hand hygiene feel manageable, in maternity wards in facilities where you have worked? [Self-efficacy]

More manageable

Less manageable

About the same

28. If you would like to, please provide an explanation for your answer:

29. Overall, how do the new campaign posters compare to the previous three posters in motivating you to practice good hand hygiene? [Perceived effectiveness]

More motivating

Less motivating

About the same

30. If you would like to, please provide an explanation for your answer:

31. What changes would you make to the new campaign posters to encourage improvements in hand hygiene in maternity wards, in facilities where you have worked? Please tick all that apply:

Text content

Volume of text

Colour scheme

Style of drawings

Patient shown

Healthcare worker shown

Other persons shown

Explanation of the benefits of good hand hygiene

No changes

Other (please specify):

32. Please select the options below that best fit your job role. Please tick all that apply:

Public Health practitioner or researcher in the field of Maternal Health

Healthcare provider or clinician with experience working in maternity settings

Infection Prevention and Control Specialist

Healthcare worker from one of 'GLOSS' participating facilities

Other:

33. Please select where you work from the dropdown menu below:

[Country dropdown list]

34. How often are the following resources used for infection control available in maternity wards, in facilities where you have worked?

A) Clean running water piped into the facility:

Always

Intermittently

Rarely

Never

35. Bucket with tap or pour pitcher providing clean running water:

Always

Intermittently

Rarely

Never

Not applicable, piped running water available

36. Handwashing-washing soap/liquid soap:

Always

Intermittently

Rarely

Never

37 Alcohol based hand rub:

Always

Intermittently

Rarely

Never

Thank you very much for your time completing this survey. Your response has been recorded. Your answers will be used to improve the acceptability and relevance of these materials for use in maternity settings, to help prevent maternal infection and sepsis.

Supplementary Material 4a: Semi-structured Interview guide investigating the acceptability of the “WHO hand hygiene reminders in the workplace” for use in maternity settings

Posters to be discussed:

Poster A – How to Handwash

Poster B – How to Handrub

Poster C – 5 Moments of Hand Hygiene

Poster D – Prevent Sepsis in Healthcare. It’s in your Hands

<u>Qualitative Interview Questions</u>

Section 1: Background, understanding and motivations

What is your job role? Where in the world have you worked?

What does it mean to you to have ‘good hand hygiene’ when working in a maternity setting?

What have your experiences around hand hygiene been in the different maternity settings you’ve worked in?

Where can you go to get information about hand hygiene and infection prevention if you had any questions or difficulties?

Section 2: Current posters and survey validation

Show posters A-C: ‘These are the WHO posters to promote and explain good hand hygiene practices’

Domain: Intervention coherence (‘the extent to which the participant understands the intervention and how it works’)

Are these posters familiar to you?

How do these posters influence your opinion of what is required for good hand hygiene in maternity settings?

PROMPT: Can you think of any situations specific to maternity settings where additional hand hygiene guidance may be helpful in these posters?

Domain: Perceived effectiveness (‘the extent to which the intervention in perceived as likely to achieve its purpose’)

What stands out to you from these posters?

Would you expect these posters to be effective at making people improve their hand hygiene in maternity settings?

Domain: Affective Attitude (‘how an individual feels about the intervention’)

How do these posters make you feel about hand hygiene?

Domain: Self Efficacy (‘the participant’s confidence that they can perform the behaviours required to participate in the intervention’)

After looking at these posters, how manageable do you think good hand hygiene is in maternity settings where you have worked?

Domain: Burden (‘the perceived amount of effort that is required to participate in the intervention’)

How much effort would it take to complete the tasks in the posters, in maternity wards where you work/have worked? Why?

Domain: Opportunity cost (‘the extent to which benefits, profits or values must be given up to engage in the intervention’)

Would following all the instructions in these posters make it difficult for you to complete other parts of your job? Why/why not?

Domain: Ethicality (‘the extent to which the intervention has good fit with an individual’s value system’)

Do you think the images in the posters are culturally appropriate to show in maternity wards where you work/have worked? Why/ why not?

If a pregnant woman was displayed in the poster would any dress requirements be needed for cultural sensitivities where you work/have worked?

Section 3: Suggestions for adaptations

Pulling all these thoughts together, can you think of any changes to these posters to make them more acceptable for maternity wards where you work/have worked?

Section 4: New poster

Show poster D: 'This a part of a new WHO campaign for good hand hygiene'

How does this poster make you feel about practicing good hand hygiene in maternity settings? If different, why is this different to the other posters?

Do you think this poster is better/worse/the same when compared to the other posters in terms of:

- Being eye catching and noticeable?
- Explaining the importance of good hand hygiene?
- Appearing relevant to maternity settings?
- Encouraging you to perform good hand hygiene practices?
- Being culturally appropriate for display in maternity settings where you have worked?

Can you think of any changes to this poster to make it more acceptable for maternity wards where you work/have worked?

Thank you! End of interview.

Supplementary Material 4b: Focus group guide investigating the acceptability of the "WHO hand hygiene reminders in the workplace" for use in maternity settings

Explain purpose of the focus group:

- WHO don't currently produce hand hygiene posters specific to maternity settings
- **Question:** Can these hand hygiene posters be adapted for use in maternity settings to improve adherence to best practice behaviours and reduce hospital acquired infections

Section 1: Background

Brief individual introductions, where participants currently work and job role

Section 2: Current posters

Show posters A-C: 'These are the WHO posters to promote and explain good hand hygiene practices'

Domain: Perceived effectiveness ('the extent to which the intervention in perceived as likely to achieve its purpose')

How can we make these posters work for maternity settings?

Do you think people working in maternity settings would take notice of these posters? Why is that?

Prompts: updating the colour scheme has been important in the survey. What colours would people suggest? Any suggestions for the style of drawings?

Domain: Intervention coherence ('the extent to which the participant understands the intervention and how it works')

How well do you think these posters explain the intervention of good hand hygiene to healthcare workers working in a maternity setting?

How can the messages in these posters be developed for maternity settings to ensure actions required are clear?

Prompts: step by step images have been important in the interviews – which further steps/images could we include?

Can you think of any situations specific to maternity settings where additional hand hygiene guidance may be helpful in these posters?

Prompts: additional scenarios to include (survey and interviews have suggested: VEs, scrubbing up, glove use), any other examples for maternity settings, idea of patient fronted component to encourage patient/guardian involvement in hand hygiene practices

Domain: Ethicality ('the extent to which the intervention has good fit with an individual's value system')

What do you think of the images in the posters at the moment?

Prompts: Showing a pregnant woman, or mother and baby has come up in the results thus far. How can we do this and still keep the posters culturally sensitive for multiple settings? What do people think of the style of the drawings?

Domain: Burden ('the perceived amount of effort that is required to participate in the intervention')

How much effort would it be for staff in your setting to complete the tasks in the posters? Why is that?

Domain: Opportunity cost ('the extent to which benefits, profits or values must be given up to engage in the intervention')

What do you think is manageable in maternity settings?

Prompt: Is the 5 moments idea still acceptable or could something else be developed? Are having numbered moments helpful or would clinical scenario based poster be more appealing?

Domain: Self Efficacy ('the participant's confidence that they can perform the behaviours required to participate in the intervention')

Are there barriers to participating fully in this intervention in your setting? Is there any way these can be addressed in the poster?

Prompts: resources, understanding, workload, motivation, time

Domain: Affective Attitude ('how an individual feels about the intervention')

Overall, how do these posters make you feel?

(Back up question if time)

Perceived effectiveness

Do you think people working in maternity settings would change their hand hygiene practices after having seen these posters? Talk me through why that is.

Prompts: Explaining the importance of hand hygiene has been important in the survey – how can we best do this?

Is there anything else you would like to say about these posters?

Prompts: colour scheme, motivation. How can motivation be enhanced? How can we make the posters appear novel but also cohesive with the overall WHO message of hand hygiene? How can we develop a positive impression from these posters for healthcare workers?

End of focus group

Supplementary Material 5: ***Map displaying countries represented by all study participants.***

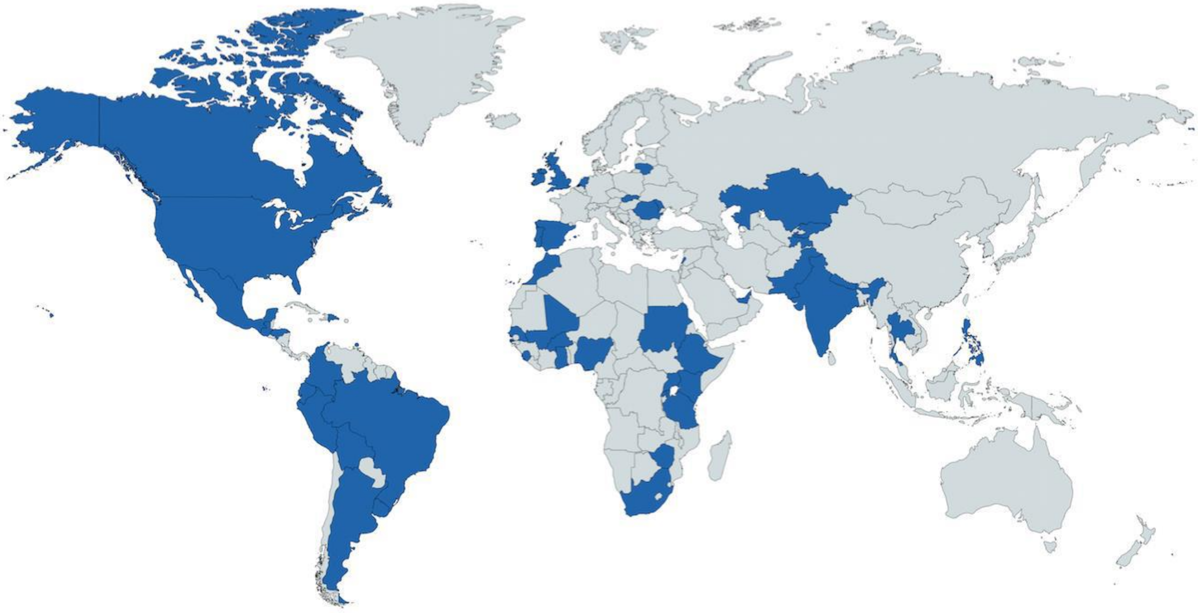

Supplementary Material 6: Hand Hygiene resources available to survey participants

Participants from LMICs stated they were less likely to have access to reliable hand hygiene infrastructure. Those from HIC had 3.74 increased odds of always have access to clean running water, when compared to those from LMIC (95% CI 1.27-10.97, P=0.016). The odds of always having access to hand hygiene resources was further increased for soap (6.26 OR (95% CI 2.17-18.05, P=0.001) and alcohol-based handrub (ABHR) (3.97 OR (95% CI 2.08 – 7.60, P<0.001).

**HAND HYGIENE RESOURCE N=263**	**FREQUENCY OF AVAILABILITY N (%)**	**High Income 76 (29%)**	**Upper Middle Income 104 (40%)**	**Lower Middle Income 57 (22%)**	**Low Income 26 (10%)**
Clean running water piped into facility	Always	72 (95)	98 (95)	41 (72)	15 (58)
	Intermittently	3 (4)	4 (4)	13 (23)	9 (35)
	Rarely	1 (1)	1 (1)	1 (2)	2 (8)
	Never	0 (0)	0 (0)	2 (4)	0 (0)
Handwashing soap/liquid soap	Always	72 (95)	82 (80)	38 (67)	18 (69)
	Intermittently	4 (5)	12 (12)	12 (21)	8 (3)
	Rarely	0 (0)	5 (5)	4 (7)	0 (0)
	Never	0 (0)	4 (4)	3 (5)	0 (0)
Alcohol based hand rub	Always	62 (82)	72 (70)	22 (39)	4 (15)
	Intermittently	12 (16)	19 (18)	19 (33)	18 (69)
	Rarely	2 (3)	7 (7)	11 (19)	4 (15)
	Never	0 (0)	5 (5)	5 (9)	0 (0)

The most commonly recorded answer by row is highlighted using the colour code of the 'frequency of availability' response. Always is depicted as green, intermittently as yellow, rarely as orange and never as red.

23% missing data for all results in this table. Country income status was classified using the World Bank listings at the time of June 2018.

Supplementary material 7: Table of hand hygiene moments

Moment	Examples in a Maternity Setting
1. Before touching a patient	Before shaking hands<br>Before measuring BP, taking vital signs or listening to the fetal heart rate<br>Before touching skin<br>Before performing bathing<br>Before feeding the patient<br>Before helping with personal care activities such as dressing the patient<br>Before a non-invasive examination activity i.e. chest auscultation
2. Before clean/aseptic procedure	Wiping the vagina<br>Vaginal Examination<br>Artificial rupture of membranes<br>Performing episiotomy<br>Catching the baby (delivering the baby)<br>Cord cutting and clamping<br>Cord traction<br>Manual removal of placenta<br>Suturing the perineum<br>Wiping the baby clean<br>Urinary catheter insertion or removal<br>Before inserting an IV line<br>Before drawing blood<br>Before preparing PO, PV, PR, IM or IV medication<br>Before handling or inserting an invasive device<br>Before touching any mucous membranes or damaged/breached skin (even if gloves are worn)<br>If moving between a contaminated body site to another body site on the same patient<br>Before physically examining a post-operative wound site<br>Before touching the wound to remove stitches/clips<br>Before preparing the necessary items for replacing the wound dressing Before replacing the actual post-operative wound dressing<br><br>Note; if the HCW stays within the patient zone between these examples they do not need to re clean hands between aseptic procedures unless Moment 3, body fluid exposure risk, occurs/is significant. Non-touch aseptic technique

	should be maintained. These moments are then considered part of the delivery ‘flow’<br>Hand hygiene is required even if gloves are worn. Hand hygiene should be performed immediately before donning gloves.
3. After body fluid exposure risk	After any task involving body fluid exposure or excretions, such as any of those mentioned in moment 2.<br>If moving between a contaminated body site to another body site on the same patient<br>After vaginal examination<br>After cutting the cord<br>After episiotomy<br>After artificial rupture of membranes<br>After delivering the baby<br>After drawing blood<br>After suturing<br>After inserting an invasive medical device (vascular access, catheter, tube, drain)<br>After handling an invasive medical device<br>After disrupting and opening an invasive circuit<br>After removing an invasive medical device<br>After removing any patient protection (napkin, dressing, gauze, sanitary towel, etc)<br>After handling an organic sample, (blood sample, placental tissue)<br>After clearing excreta, blood, urine or amniotic fluid<br>After cleaning any contaminated surface and soiled material (soiled bed linen, dentals, instruments, urinal, bedpan, lavatories)
4. After touching a patient	After shaking hands<br>After measuring BP, taking vital signs or listening to the fetal heart rate<br>After touching skin<br>After performing bathing<br>After touching the patient’s immediate surroundings<br>After a non-invasive examination activity i.e. chest auscultation
5. After touching patient surroundings	After any touch in the patient zone of patient surroundings
Additional notes:	Wear gloves if contact with body fluids is anticipated; the need for hand hygiene does not change even if gloves are worn, as per the WHO 5 Moments.

	Use Aseptic technique, including non-touch technique for all aseptic procedures.<br><br>If gloves are being worn then hand hygiene should be performed immediately before they are put on, and immediately after removal to avoid recontamination.
