# Supplementary material for: Adapting the WHO hand hygiene ‘reminders in the workplace’ to improve acceptability for healthcare workers in maternity settings worldwide: a mixed methods study
Source: BMJ Open. 2024 Sep 17;14(9):e083132. doi: 10.1136/bmjopen-2023-083132 (PMC11409321; doi:10.1136/bmjopen-2023-083132)
Supplement: Supplementary file 2 [file bmjopen-2023-083132.reviewer_comments.pdf]

PEER REVIEW HISTORY

BMJ Open publishes all reviews undertaken for accepted manuscripts. Reviewers are asked to complete a checklist review form and are provided with free text boxes to elaborate on their assessment. These free text comments are reproduced below.

ARTICLE DETAILS

**TITLE (PROVISIONAL)**	Adapting the WHO Hand Hygiene "Reminders in the Workplace" to Improve Acceptability for Healthcare Workers in Maternity Settings Worldwide: a Mixed Methods Study
**AUTHORS**	Dunlop, Catherine; Kilpatrick, Claire; Jones, Laura; Bonet, Mercedes; Allegranzi, B; Brizuela, Vanessa; Graham, Wendy; Thompson, Amy; Cheshire, James; Lissauer, David

VERSION 1 - REVIEW

**REVIEWER NAME**	kasem, abedallah
**REVIEWER AFFILIATION**	Jordan University of Science and Technology, maternal and child health department faculty of nursing
**REVIEWER CONFLICT OF INTEREST**	THERE IS NO CONFLICT OF INTEREST WORTH OF MENTION
**DATE REVIEW RETURNED**	31-Dec-2023

**GENERAL COMMENTS**	the researchers made a great effort in constructing this mixed methods study. They also collected data from different participants in different countries. However, when it comes to mixed methods design, I would suggest that the table must be presented easily and separately. for example, table 2 provided mixed presentations of both qualitative and quantitative data and I believe this is confusing. although the statistical test describing quantitative data is not clearly defined and was not linked clearly to the aim and objectives of the study.

**REVIEWER NAME**	Kumar, Praveen
**REVIEWER AFFILIATION**	Post Graduate Institute of Medical Education and Research, Pediatrics
**REVIEWER CONFLICT OF INTEREST**	None
**DATE REVIEW RETURNED**	07-Jan-2024

**GENERAL COMMENTS**	General comments<br>The manuscript systematically evaluates the acceptability of WHO Hand Hygiene workplace reminder posters in maternity care settings, which is pertinent given the unique challenges and high risk of healthcare associated infections (HCAI) associated with maternal-newborn care. The study methodology, analysis, and interpretation are robust, providing valuable insights into the development of the new WHO posters and barriers related to hand hygiene.<br>Strengths:<br>• The study highlights key concepts for designing effective posters, emphasizing preferences for pictorial representations, minimal text,

	context relevance, and clear indications of why healthcare workers should follow specific instructions.<br>• The manuscript provides a comprehensive understanding of background processes involved in poster development, offering insights into barriers related to hand hygiene.<br>Specific comments and suggestions<br>• Consider moving Figure 1 to supplementary material or simply provide a weblink.<br>• Table 2: Define Good and Excellent scores.<br>• Survey participants: Although the survey spanned 51 countries and 342 participants, the representativeness is skewed, possibly because of the survey sampling strategy. The bulk of HCAI occur in LMIC, however the representation of African and South Asian countries was less. Also, the respondents from Asian and African countries could have had difficulty in interpretation of questions and expression of responses due to English being the primary medium of the survey. In fact, Spanish was the most used language in the survey. Only 166/342 survey participants were HCWs with experience of working in maternity settings. It may be useful to see if the survey results were similar for these HCW vs. other expert participants. It looks like a single focus group involving 7 participants was conducted, which may not be enough to saturate the themes. Clarify if the interviews and focused group discussions (FGD) were conducted in person or online.<br>• Issues in resource constrained settings: Though the primary objective was the development of new setting-specific reminders, the study does highlight the well-known fact that resource constraints in LMICs are a significant barrier to actual implementation of hand hygiene practices. The authors do bring out that the respondents from LMICs felt that reminders weren't reflective of the realities of resource limited settings in LMIC. Another very pertinent issue in LMICs is overcrowding with bed-sharing and minimal space between beds, blurring the boundaries of 'patient zones' (1). The posters developed based on a single patient per bed with adequate space between the beds are difficult to relate to, by the HCWs working in these environments. What constitutes a hand hygiene opportunity in the bed-sharing ward is difficult to understand.<br>• Exclusion of patients and public: This study focused on HCW's perceptions, and patients or public were not included. Hand Hygiene is one tool which is common to HCW, as well as patients and visitors. In the era of family cantered care, the patients and visitors are equal partners. Post-Covid, even the lay public has become aware of the importance and methods of hand hygiene. So, it is better to have a common set of posters directed towards HCW, patients and visitors. Hence, it would have been useful if patients and public were also included among those surveyed.<br>• Effectiveness of reminders: Though reminder posters are a useful strategy to influence behaviour, they quickly become 'invisible' as was found in the responses from HIC in the current study. They need to be frequently changed in their 'look' or 'colour' to make them noticed again. The WHO hand hygiene self-assessment framework suggests auditing the reminders for damage and replacement every 2-3 months. Though the exact frequency of change is not known, monthly reminders were noted to be more effective than quarterly reminders to sustain practice change among direct care providers in residential care facilities (2). The authors rightly acknowledge that reminders are only one part of the wider WHO multimodal intervention strategy. They have only a moderate success in bringing about behaviour change. We need to innovative ideas

	under the categories of simplification, automation and forcing functions.<br>• Post-evaluation: The final developed posters have not been evaluated for their acceptability or effectiveness. The authors do emphasize that the impact of the new posters on hand hygiene compliance should be evaluated in varying settings.<br>References<br>1. Salmon S, McLaws ML. Environmental challenges of identifying a patient zone and the healthcare zone in a crowded Vietnamese hospital. J Hosp Infect. 2015 Sep;91(1):45-52. doi: 10.1016/j.jhin.2015.04.020.<br>2. Slaughter, S.E., Eliasziw, M., Ickert, C. et al. Effectiveness of reminders to sustain practice change among direct care providers in residential care facilities: a cluster randomized controlled trial. Implementation Sci 2020; 15, 51. https://doi.org/10.1186/s13012-020-01012-z

VERSION 1 – AUTHOR RESPONSE

Reviewer: 1
Dr. Hussien AL-qasem, Zarqa Private University Faculty of Allied Medical Sciences, Zarqa Private University. Comments to the Author:

Reviewer 1 comments	Author response
The researchers made a great effort in constructing this mixed methods study. They also collected data from different participants in different countries.	Thank you for your positive feedback regarding our study
However, when it comes to mixed methods design, I would suggest that the table must be presented easily and separately. For example, table 2 provided mixed presentations of both qualitative and quantitative data and I believe this is confusing.	Thank you for your feedback on table 2. We appreciate mixed methods studies are uncommon in the scientific literature, but based on best practice guidance for mixed methods research, a joint display is the optimal way of presenting results for this methodology (1). This is because the joint display helps emphasise the integration stage of data analysis, combining the findings from the quantitative and qualitative data, which is crucial in mixed methods research. This is why we have chosen to combine the quantitative and qualitative results for each construct in the same table (1).<br><br>For these reasons our preference would be to keep the quantitative and qualitative results in the same table as a joint display. We have renamed the table 'Joint display of Mixed Methods results for the Three Central Hand Hygiene Reminders' to reduce confusion. A further statement about presenting in a joint

	display has been added to the data integration section of the methods.<br><br>(1)Guetterman TC, Fetters MD, Creswell JW. Integrating Quantitative and Qualitative Results in Health Science Mixed Methods Research Through Joint Displays. Ann Fam Med. 2015 Nov;13(6):554-61. doi: 10.1370/afm.1865. PMID: 26553895; PMCID: PMC4639381.
Although the statistical test describing quantitative data is not clearly defined and was not linked clearly to the aim and objectives of the study.	Details about the test have been added to the methods section and the rationale explained.

Reviewer: 2
Dr. Praveen Kumar, Post Graduate Institute of Medical Education and Research
Comments to the Author:

Comments to Author	Author Response
General comments<br>The manuscript systematically evaluates the acceptability of WHO Hand Hygiene workplace reminder posters in maternity care settings, which is pertinent given the unique challenges and high risk of healthcare associated infections (HCAI) associated with maternal-newborn care. The study methodology, analysis, and interpretation are robust, providing valuable insights into the development of the new WHO posters and barriers related to hand hygiene.	Thank you for your positive feedback regarding our study
Strengths:<br>• The study highlights key concepts for designing effective posters, emphasizing preferences for pictorial representations, minimal text, context relevance, and clear indications of why healthcare workers should follow specific instructions.<br>• The manuscript provides a comprehensive understanding of background processes involved in poster development, offering insights into barriers related to hand hygiene.	We appreciate the time you have taken for specific and thoughtful comments based on a thorough read of our manuscript.

Specific comments and suggestions<br>• Consider moving Figure 1 to supplementary material or simply provide a weblink.	Figure 1 has been moved to supplementary materials
• Table 2: Define Good and Excellent scores.	Thank you for raising this. A definition has been included below the table and details added into the text of the manuscript.
• Survey participants: Although the survey spanned 51 countries and 342 participants, the representativeness is skewed, possibly because of the survey sampling strategy. The bulk of HCAI occur in LMIC, however the representation of African and South Asian countries was less. Also, the respondents from Asian and African countries could have had difficulty in interpretation of questions and expression of responses due to English being the primary medium of the survey. In fact, Spanish was the most used language in the survey.	Thank you for this point. We have added a note in potential limitations related to lack of availability of the survey in other languages. However, we were pleased to be able to provide the survey in four languages, which were the most commonly spoken languages for the countries that participated in the GLOSS study (our survey sample).<br><br>However, the countries included showed more of a skew towards including LMICs, where 187 participants were included, as compared to 76 from HICs. Therefore, we hope that the survey results do reflect the importance of the LMIC perspective on HCAI, given it's predominance in this setting.
Only 166/342 survey participants were HCWs with experience of working in maternity settings. It may be useful to see if the survey results were similar for these HCW vs. other expert participants.	Unfortunately, it is not possible for us to run this analysis meaningfully, as participants were able to select more than one job role in the survey. However, as well as the 166 who were HCWs with experience of working in maternity settings, there were also 100 people that selected their role as a healthcare worker in GLOSS participating facilities. This means these participants would have also had a clinically facing role in a maternity setting. Therefore, it is likely that the majority of the participants did have experience of clinical work in maternity settings.
It looks like a single focus group involving 7 participants was conducted, which may not be enough to saturate the themes.	Thank you for this point. The purpose of the focus group was to explore and elaborate on the themes already arising from the qualitative interviews, in order to practically consider how these could be applied to new reminders for the workplace. The qualitative interviews had already been continued until data adequacy was reached. These details have now been included in the manuscript for clarity.

Clarify if the interviews and focused group discussions (FGD) were conducted in person or online.	This detail has been added. They were conducted face to face.
Issues in resource constrained settings: Though the primary objective was the development of new setting-specific reminders, the study does highlight the well-known fact that resource constraints in LMICs are a significant barrier to actual implementation of hand hygiene practices. The authors do bring out that the respondents from LMICs felt that reminders weren't reflective of the realities of resource limited settings in LMIC.	Thank you for noting this point and our findings surrounding this.
Another very pertinent issue in LMICs is overcrowding with bed-sharing and minimal space between beds, blurring the boundaries of 'patient zones' (1). The posters developed based on a single patient per bed with adequate space between the beds are difficult to relate to, by the HCWs working in these environments. What constitutes a hand hygiene opportunity in the bed-sharing ward is difficult to understand.<br><br>1. Salmon S, McLaws ML. Environmental challenges of identifying a patient zone and the healthcare zone in a crowded Vietnamese hospital. J Hosp Infect. 2015 Sep;91(1):45-52. doi: 10.1016/j.jhin.2015.04.020.	Thank you for noting this important point. We agree that overcrowding can be a huge issue, especially in LMICs, that impacts severely on effective infection prevention and control. The WHO guidance is still to avoid bed sharing between different adult patients in maternity settings in order to reduce the risk of the spread of infection. However, for microbiological purposes the mother and her newborn baby remain a part of the same patient zone as it applies to hand hygiene action, therefore it is acceptable from an infection prevention and control perspective for these patients to share the same bed. The new hand hygiene reminders were drawn with the mother and infant in the same 'patient zone' to reflect this. We have added this detail to the manuscript, and additionally included this reference.
• Exclusion of patients and public: This study focused on HCW's perceptions, and patients or public were not included. Hand Hygiene is one tool which is common to HCW, as well as patients and visitors. In the era of family cantered care, the patients and visitors are equal partners. Post-Covid, even the lay public has become aware of the importance and methods of hand hygiene. So, it is better to have a common set of posters directed towards HCW, patients and visitors. Hence, it would have been useful if patients and public were also included among those surveyed.	Thank you for this point. We agree that the patient and visitor perspective is very important in hand hygiene research and effective implementation. The healthcare workers desire to involve patients in hand hygiene was one of the components that developed qualitatively in the 'other' theme, which is already described in the results section of our manuscript.<br><br>We have added this as a limitation in the discussion and included a recommendation that the patient perspective be sought on future research of the hand hygiene reminders. It is however, worth noting that the WHO 5 Moments posters are targeted at healthcare workers not

	patients and as such patients were not the target audience for this study.<br><br>We have added clarification in the title of the study that our research question was specifically looking at the acceptability of the hand hygiene reminders for healthcare workers in maternity settings.
• Effectiveness of reminders: Though reminder posters are a useful strategy to influence behaviour, they quickly become 'invisible' as was found in the responses from HIC in the current study. They need to be frequently changed in their 'look' or 'colour' to make them noticed again. The WHO hand hygiene self-assessment framework suggests auditing the reminders for damage and replacement every 2-3 months. Though the exact frequency of change is not known, monthly reminders were noted to be more effective than quarterly reminders to sustain practice change among direct care providers in residential care facilities (2).<br><br>2. Slaughter, S.E., Eliasziw, M., Ickert, C. et al. Effectiveness of reminders to sustain practice change among direct care providers in residential care facilities: a cluster randomized controlled trial. Implementation Sci 2020; 15, 51. https://doi.org/10.1186/s13012-020-01012-z	Thank you for this point. As you mention, our participants agreed, and this is currently presented in the results and discussed in the discussion section. Additionally, we discuss the recommendation for audit and damage replacement every 2-3 months in the discussion section.<br><br>We have added to the discussion the additional point that monthly reminders were more effective than quarterly reminders to sustain practice in residential care settings, with the reference. Thank you for bringing this to our attention.
The authors rightly acknowledge that reminders are only one part of the wider WHO multimodal intervention strategy. They have only a moderate success in bringing about behaviour change. We need to innovative ideas under the categories of simplification, automation and forcing functions.	Thank you for this point. As you mention, we already have a paragraph detailing this in the discussion and the literature around MMIS was reviewed for relevance to this study/paper. We have added a further sentence regarding the potential for evaluating other options and innovations in hand hygiene to improve hand hygiene compliance alongside the MMIS.
• Post-evaluation: The final developed posters have not been evaluated for their acceptability or effectiveness. The authors do emphasize that the impact of the new posters on hand hygiene compliance should be evaluated in varying settings.	Thank you for this point, and we fully agree. As you mention, it is already emphasized in the discussion section that the new reminders need to be evaluated to understand their effectiveness.

VERSION 2 – REVIEW

**REVIEWER NAME**	kasem, abedallah
**REVIEWER AFFILIATION**	Jordan University of Science and Technology, maternal and child health department faculty of nursing
**REVIEWER CONFLICT OF INTEREST**	there is no conflict of interest
**DATE REVIEW RETURNED**	22-Apr-2024

**GENERAL COMMENTS**	the authors did their best to response to the reviewers comment.<br>I still believe that the qualitative data must be presented in separate table and not in one table. their is a miss match between the qualitative data presented in table 2 to support intervention coherence. for example " "I think that everyone would know what to do when<br>they see the posters. Like I said the message is clear,<br>so reading through that and with the pictures attached<br>everyone would know when to wash hands and how to<br>do it." P2 Interviewee (LMIC)"<br>what is the table 2 could not be find in the text .<br><br>the discussion part is still in need for good presentation as it was described in brief and doesn't reflect both quantitative and qualitative. I advise the authors to present discussion for each separately.

**REVIEWER NAME**	Kumar, Praveen
**REVIEWER AFFILIATION**	Post Graduate Institute of Medical Education and Research, Pediatrics
**REVIEWER CONFLICT OF INTEREST**	No competing interests
**DATE REVIEW RETURNED**	21-Apr-2024

**GENERAL COMMENTS**	Thank you for the clarifications to the queries and the changes. Congratulations on the excellent work !<br><br>P.S. Regarding the difficulty in defining patient zones in LMIC, the hospitals are forced to keep more than one woman or sick neonate in one bed or cot due to a mismatch between the demand and supply, despite guidelines to the contrary. The mother and her baby, of course, are and should be treated as a single unit and are part of the same patient zone.

VERSION 2 – AUTHOR RESPONSE

Reviewer: 1
Dr. abedallah kasem, Jordan University of Science and Technology, Jordan University of Science and Technology
Comments to the Author:
the authors did their best to response to the reviewers comment.

I still believe that the qualitative data must be presented in separate table and not in one table.

Response:

For this mixed methods study, we have followed standard methods and processes for collecting, analysing and "mixing" the quantitative and qualitative results into an integrated analysis[1], and therefore, present the results as such. In utilizing this design, the authors fully understood both quantitative and qualitative research methods and the process of then presenting the overall integrated findings as a 'mixed method' study. The convergent mixed methods process used is clearly described in our methods section of this paper.

This was not a multi methods study where results are typically presented separately. As such, we do not believe the joint display table identified should be changed to present the results separately under quantitative and qualitative headings, as this would not meet good practice criteria for the mixed methods approach that we have used.[2]

Their is a miss match between the qualitative data presented in table 2 to support intervention coherence. For example " "I think that everyone would know what to do when they see the posters. Like I said the message is clear, so reading through that and with the pictures attached everyone would know when to wash hands and how to do it." P2 Interviewee (LMIC)". what is the table 2 could not be find in the text .

Response: thank you for highlighting this point. The aim was to present different results in the table and then in the text. This is normal for presenting these types of results, therefore, the text remains the same.

The discussion part is still in need for good presentation as it was described in brief and doesn't reflect both quantitative and qualitative. I advise the authors to present discussion for each separately.

Response: thank you for this comment. The discussion section has been updated to better reflect the results, featuring some of the exact quantitative figures. However, as noted in the previous response regarding mixed methods, we have not fully separated out the discussion between quantitative and qualitative results as this would not be appropriate for the type of study, but have addressed both aspects more clearly throughout the current structure.

Reviewer: 2
Dr. Praveen Kumar, Post Graduate Institute of Medical Education and Research
Comments to the Author:
Thank you for the clarifications to the queries and the changes. Congratulations on the excellent work !

---

[1] Creswell J, Plano-Clark V. Designing & Conducting Mixed Methods Research. Third Edition. Sage Publications. 2017
[2] Fetters, M. D., & Tajima, C. (2022). Joint Displays of Integrated Data Collection in Mixed Methods Research. International Journal of Qualitative Methods, 21. https://doi.org/10.1177/16094069221104564

Response: thank you very much for your response and for the time taken to review and comment on this study.

P.S. Regarding the difficulty in defining patient zones in LMIC, the hospitals are forced to keep more than one woman or sick neonate in one bed or cot due to a mismatch between the demand and supply, despite guidelines to the contrary. The mother and her baby, of course, are and should be treated as a single unit and are part of the same patient zone.

Response: thank you very much again for this comment. We agree and will continue to promote this approach to support safe mother and baby care.

Changes/page number	Reason
Word added to abstract (results) – page 3	To ensure clarity with the construct as per the formal wording used in table 2 (to avoid any confusion with presenting results)
Word added to abstract (results) – page 3	To explain which construct the findings fell within
Superfluous words removed (results) – page 3	To keep within word limit
Words removed and added - Strengths and limitations – page 4	As per editor instruction as for consistency with these sections (x2 places within the article)
Words added – Results - page 11	Based on reviewer 2 comments, words added to ensure consistency with terminology across the constructs
Text added – Discussion – page 22	Text added to address reviewer 2 comments and make the discussion consistent with the results text
Text added – Discussion – page 23	Text added to address reviewer 2 comments and make the discussion consistent with the results text
Text added – Discussion – page 24	Words added to link the discussion here to the relevant construct – clarity for the reader
Text added – Discussion – page 25	Words added to link the discussion here to the relevant construct – clarity for the reader
